# Effects of exogenous thymosin β4 on carbon tetrachloride-induced liver injury and fibrosis

**DOI:** 10.1038/s41598-017-06318-5

**Published:** 2017-07-19

**Authors:** Xiankui Li, Lei Wang, Cai Chen

**Affiliations:** 1 Department of Biochemistry and Molecular Biology, School of Basic Medical Science, Tianjin Medical University, Tianjin, China; 2 Department of Respiratory Medicine, First Affiliated Hospital of Zhengzhou University, Zhengzhou, China; 3 Teaching and Research Centre, Faculty of Medicine, Xinyang Vocational and Technical College, Xinyang, China

## Abstract

The present study investigated the effects of exogenous thymosin β4 (TB4) on carbon tetrachloride (CCl_4_)-induced acute liver injury and fibrosis in rodent animals. Results showed that both in mice and rats CCl_4_ rendered significant increases in serum alanine aminotransferase and aspartate aminotransferase, hepatic malondialdehyde formation, decreases in antioxidants including superoxide dismutase and glutathione, and up-regulated expressions of transforming growth factor-β1, α-smooth muscle actin, tumor necrosis factor-α and interleukin-1β in the liver tissues. Hydroxyproline contents in the rat livers were increased by CCl_4_. Histopathological examinations indicated that CCl_4_ induced extensive necrosis in mice livers and pseudo-lobule formations, collagen deposition in rats livers. However, all these changes in mice and rats were significantly attenuated by exogenous TB4 treatment. Furthermore, up-regulations of nuclear factor-κB p65 protein expression by CCl_4_ treatment in mice and rats livers were also remarkably reduced by exogenous TB4 administration. Taken together, findings in this study suggested that exogenous TB4 might prevent CCl_4_-induced acute liver injury and subsequent fibrosis through alleviating oxidative stress and inflammation.

## Introduction

Thymosin β4 (TB4) is a small 5 kD acidic peptide, originally isolated from calf thymus and identified as the main intracellular G-actin sequestering peptide in cell^1, 2^. It harbors multiple functions and is involved in many important pathophysiological processes, including angiogenesis^3, 4^, wound healing and repair^5, 6^, inflammation^7–9^ and cancer progression^10–12^.

In the past several decades many investigations have been done to study the physiological benefits of exogenous TB4 in the body. For example, exogenous TB4 administration enhances skin^6^ and corneal^13^ wound healing in mice by promoting keratinocyte migration and inhibiting inflammation^6, 13^. Exogenous TB4 treatment also provides cardio-protection against ischemic injury in mice through promoting cardiac cell migration, survival and reprogramming epicardial cells into cardiomyocytes^14–19^. However, since TB4 is ubiquitously expressed throughout the body^20^, the potential physiological functions of TB4 might be far more than current findings and needs further explorations. Recently, TB4 was investigated in the liver^21^. Clinical study showed that in patients with liver diseases the serum TB4 levels were negatively correlated with the liver function^22^. *In vitro* cell culture experiments demonstrated that TB4 treatment increased hepatic growth factor production and decreased PDGF-β receptor expression in hepatic stellate cells (HSCs)^23^; *in vitro* proliferation of human hepatocytes was promoted by exogenous TB4 treatment^24^. Exogenous TB4 administration also ameliorated ischemia reperfusion-induced hepatic injury in mice through activation of AKT-Bad signaling pathway^25^. In the liver tissues of carbon tetrachloride (CCl_4_)-treated rats TB4 mRNA levels were increased^26^. Reyes-Gordillo and his colleagues reported that exogenous TB4 treatment prevented the histological appearances of necrosis, inflammatory infiltration, up-regulations of α1(and 2) collagen, α-SMA, PDGF-β receptor and fibronectin mRNA expressions, and maintained quiescent phenotypic state of hepatic stellate cells in the liver tissues of CCl_4_-treated rats^27^. All these findings suggested that TB4 harbored hepatoprotective effects and might exert antifibrotic activities *in vivo*. Therefore, the main purposes of our present study were to confirm the hepatoprotective effects of exogenous TB4 against CCl_4_-induced acute mouse liver injury and investigate the *in vivo* antifibrotic activities of exogenous TB4 in the CCl_4_-induced rat liver fibrosis models.

## Results

### Acute liver injury

#### TB4 protected against CCl_4_-induced acute hepatic dysfunction

**Figure 1:**
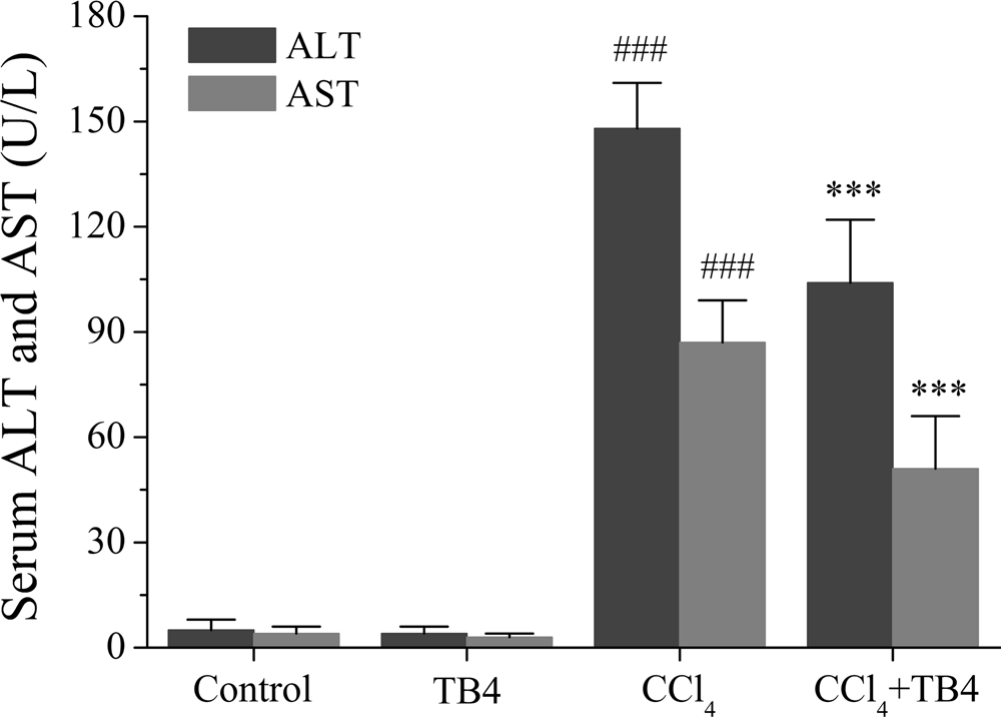
Effect of exogenous thymosin β4 (TB4) on serum alanine aminotransferase (ALT) and aspartate aminotransferase (AST) activities of mice in CCl_4_-induced hepatotoxicity. ^###^P < 0.001 vs. both control group and TB4 group; ***P < 0.05 vs. CCl_4_ group. (n = 10 for each group).

Activities of serum ALT and AST were measured to determine the effects of TB4 on the liver damage in CCl_4_-treated mice (Fig. 1). In CCl_4_-treated mice the activities of serum ALT and AST were markedly increased as compared with those in mice treated with saline (control group) or TB4 (TB4 group) (P < 0.001 for both group). Interestingly, treatment with TB4 significantly reduced the serum ALT and AST activities in CCl_4_-treated mice (P < 0.05).

#### TB4 alleviated CCl_4_-induced histological changes in the livers

**Figure 2:**
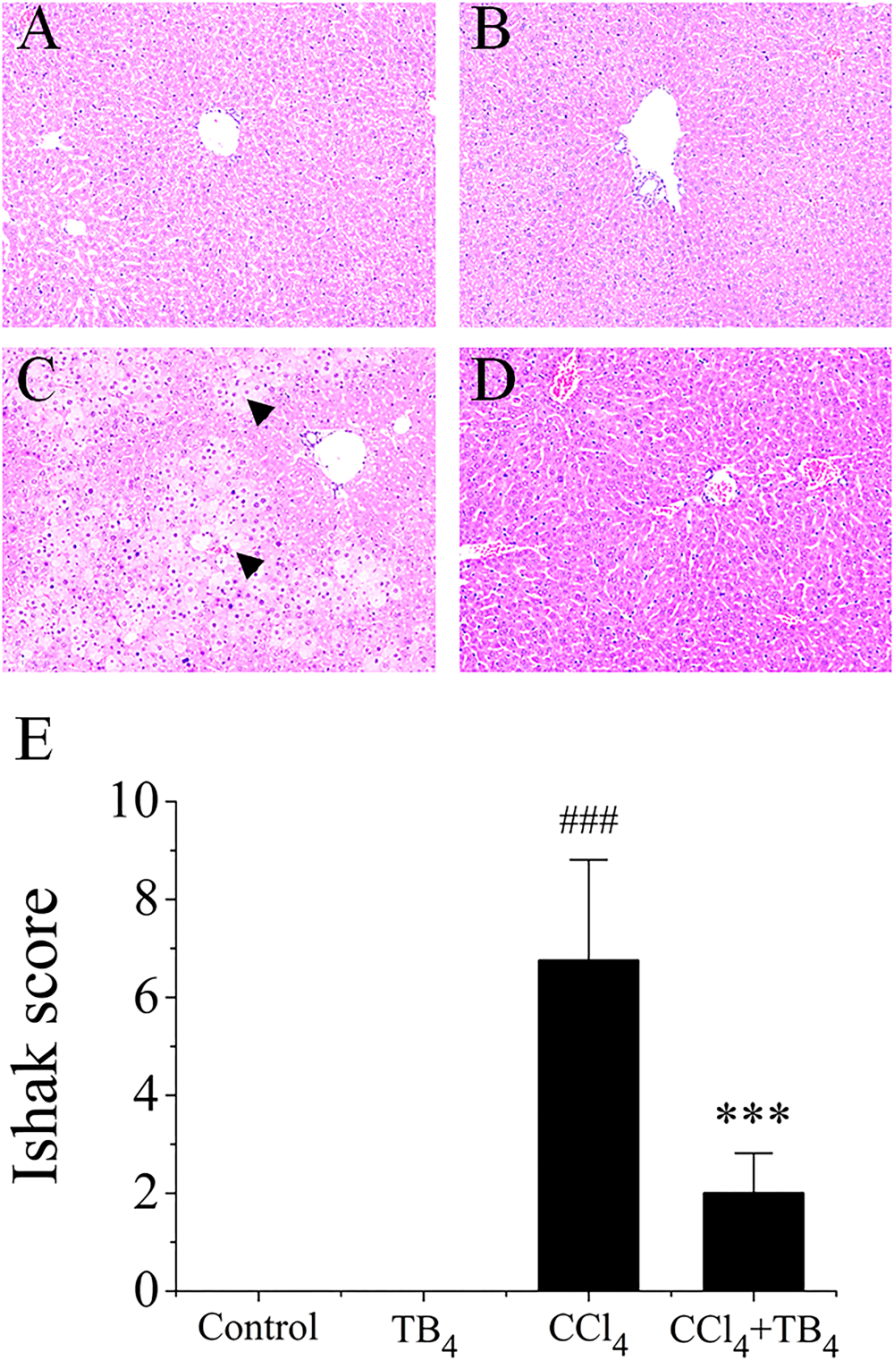
Effect of exogenous thymosin β4 (TB4) on liver histology in CCl_4_-treated mice. Representative microphotographs of liver histology staining of hematoxylin-eosin (H.E) were shown (original magnification, × 100). (**A**) Control group; (**B**) TB4 group; (**C**) CCl_4_ group; Arrow heads indicated the necrotic area. (**D**) CCl_4_ + TB4 group. (**E**) Quantitative analysis of the liver injury using Ishak scoring system. Data were expressed as mean ± standard deviation (SD). Differences were compared using Student *t*-test. ^###^P < 0.05 *vs* Control group and TB4 group; ***P < 0.05 *vs* CCl_4_ group. (n = 10 for each group).

Effects of TB4 on CCl_4_-induced liver injury in mice were investigated through histology studies. As shown in Fig. 2, livers from control group and TB4 group exhibited normal histological morphologies (Fig. 2A and B), while livers from CCl_4_-treated mice presented extensive hepatocellular necrosis (Fig. 2C). However, TB4 treatment markedly ameliorated CCl_4_-induced damages in the mouse livers (Fig. 2D). Semi-quantitative analysis of the histopathological changes using Ishak scoring system also indicated the significant protection of TB4 on CCl_4_-induced acute liver injury (Fig. 2E). These histopathological analysis results were consistent with the serum diagnostic test reports.

#### TB4 inhibited CCl_4_-induced oxidative stress and inflammation in the livers

**Figure 3:**
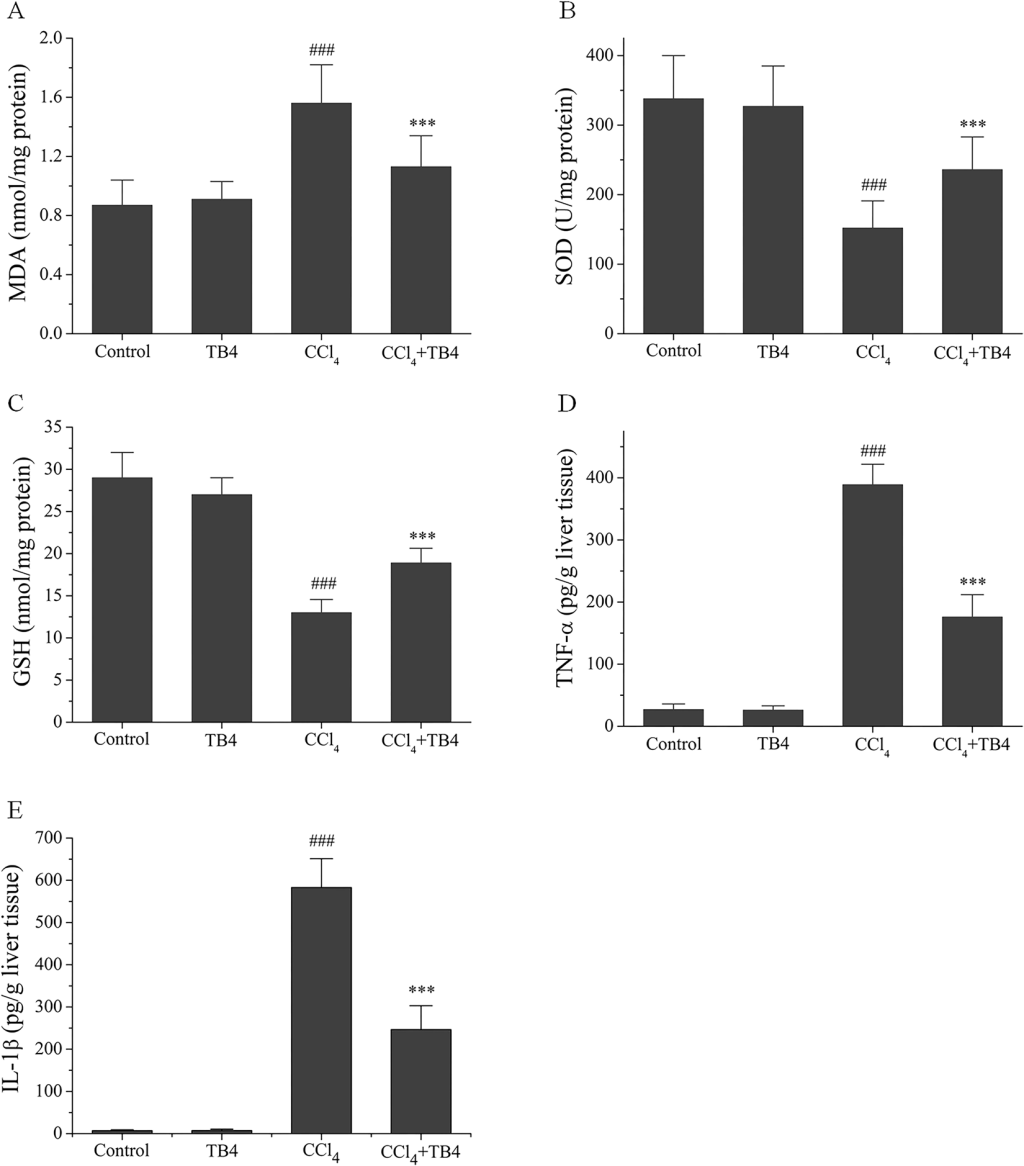
Effect of exogenous thymosin β4 (TB4) on oxidative stress parameters (**A**–**C**) and inflammatory cytokine TNF-α (**D**) and IL-1β (**E**) in the livers of CCl_4_-treated mice. ^###^P < 0.01 vs. both control group and TB4 group; ***P < 0.05 vs. CCl_4_ group. (n = 10 for each group).

**Figure 4:**
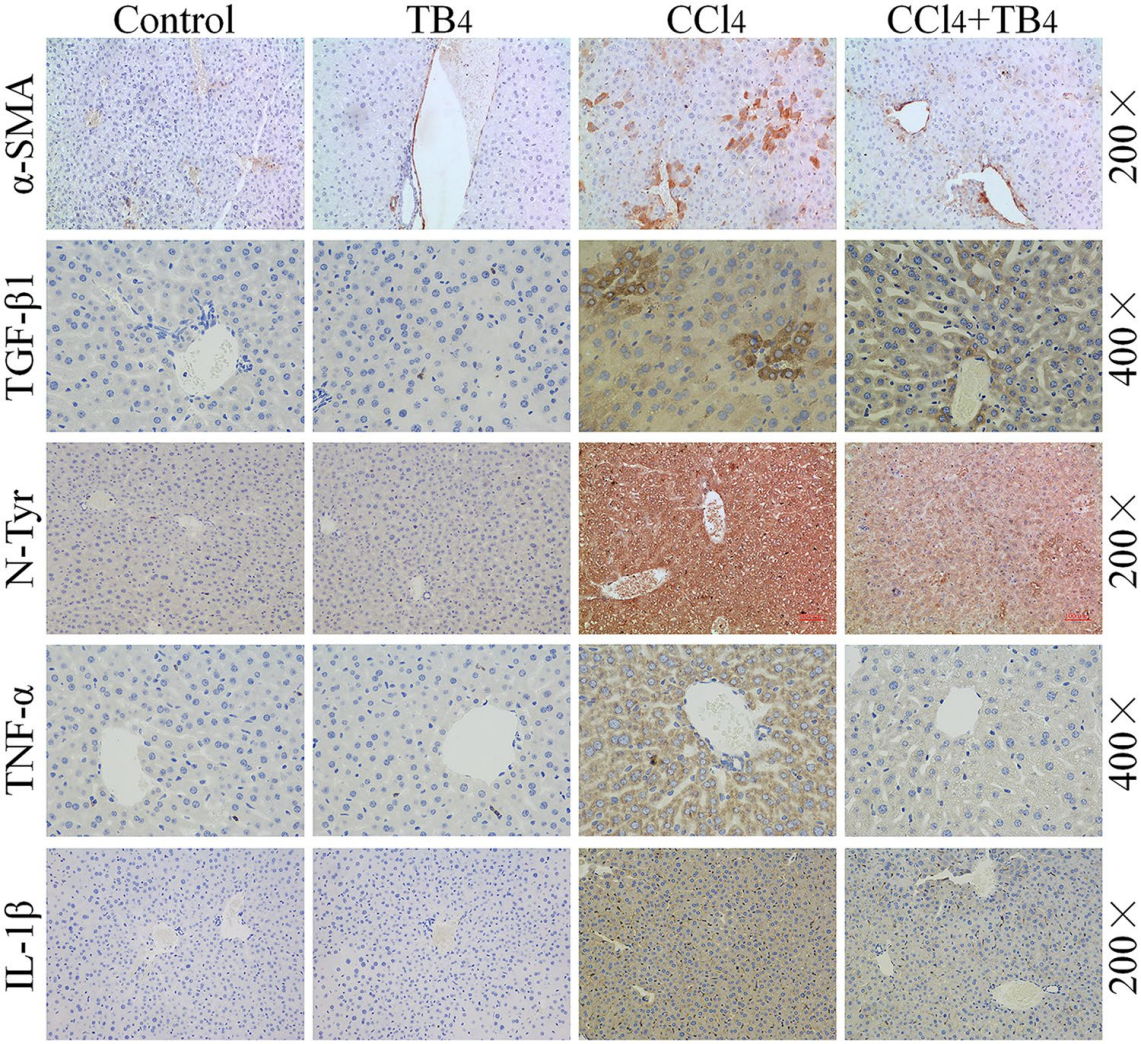
Immunohistochemistry of α-SMA, TGF-β1, nitrative tyrosine, TNF-α and IL-1β in liver sections from different groups with treatment as indicated in the figures. Original magnification of the microphotographs was showed in the figure. Four liver sections were randomly selected from each group and used for immunohistochemistry analysis. Here showed the representative results of immunohistochemistry.

Oxidative stress was assessed by determining the MDA levels, protein tyrosine nitration (Nitro-Tyrosine, N-Tyr) and the anti-oxidation activities in the liver tissues. As shown in Fig. 3A, MDA levels were significantly increased in CCl_4_-treated mice as compared with those in control and TB4-treated mice (P < 0.01). What’s more, immunohistochemistry analyses indicated that N-Tyr levels in the mouse liver tissues were also markedly up-regulated by CCl_4_ treatment (Fig. 4). However, TB4 administration significantly lowered the MDA and N-Tyr levels in the livers of CCl_4_-treated mice (Figs 3A and 4).

Anti-oxidation activities were evaluated by measuring SOD activities and GSH levels in the liver tissues. As demonstrated in Fig. 3B and C, CCl_4_ treatment significantly decreased SOD activities and GSH levels as compared with control group and TB4 group (P < 0.01 for both). However, CCl_4_-reduced SOD activities and GSH levels in the liver tissues were markedly reversed by TB4 administration (P < 0.05).

CCl_4_-induced inflammation in the liver tissue was assessed by determining the levels of pro-inflammatory cytokines including TNF-α and IL-1β. As shown in Fig. 3D and E, TNF-α and IL-1β levels in the livers were markedly increased in CCl_4_-treated mice as compared with control mice and TB4 alone treated mice (P < 0.01). However, TB4 treatment significantly decreased the TNF-α and IL-1β levels in the liver tissues of CCl_4_-treated mice (P < 0.05). Effects of TB4 on TNF-α and IL-1β expression in the liver tissues of CCl_4_-treated mice were also reconfirmed by immunohistochemistry assays (Fig. 4). Immunohistochemistry results showed that both TNF-α and IL-1β expressions in the livers were markedly increased by CCl_4_ treatment, which was greatly reduced by TB4 administration (Fig. 4).

#### TB4 inhibited CCl_4_-induced HSCs activation and reduced TGF-β1 expression in the livers

Immunohistochemistry assays demonstrated that more α-SMA-positive cells in the liver tissues were seen in CCl_4_-treated mice as compared with control mice and TB4 alone treated mice (Fig. 4), which indicated that CCl_4_ activated HSCs *in vivo*. However, up-regulation of α-SMA-positive cells in the liver tissues by CCl_4_ treatment was greatly reduced by TB4 administration. TGF-β1, a pro-fibrotic cytokine, was also up-regulated in the liver tissues of CCl_4_-treated mice, which was also reduced by TB4 administration (Fig. 4). These results suggested that exogenous TB4 administration might exhibit anti-fibrotic activities in the mouse livers.

#### TB4 suppressed CCl_4_-increased nuclear factor-κB (NF-κB) p65 protein expression in the livers

**Figure 5:**
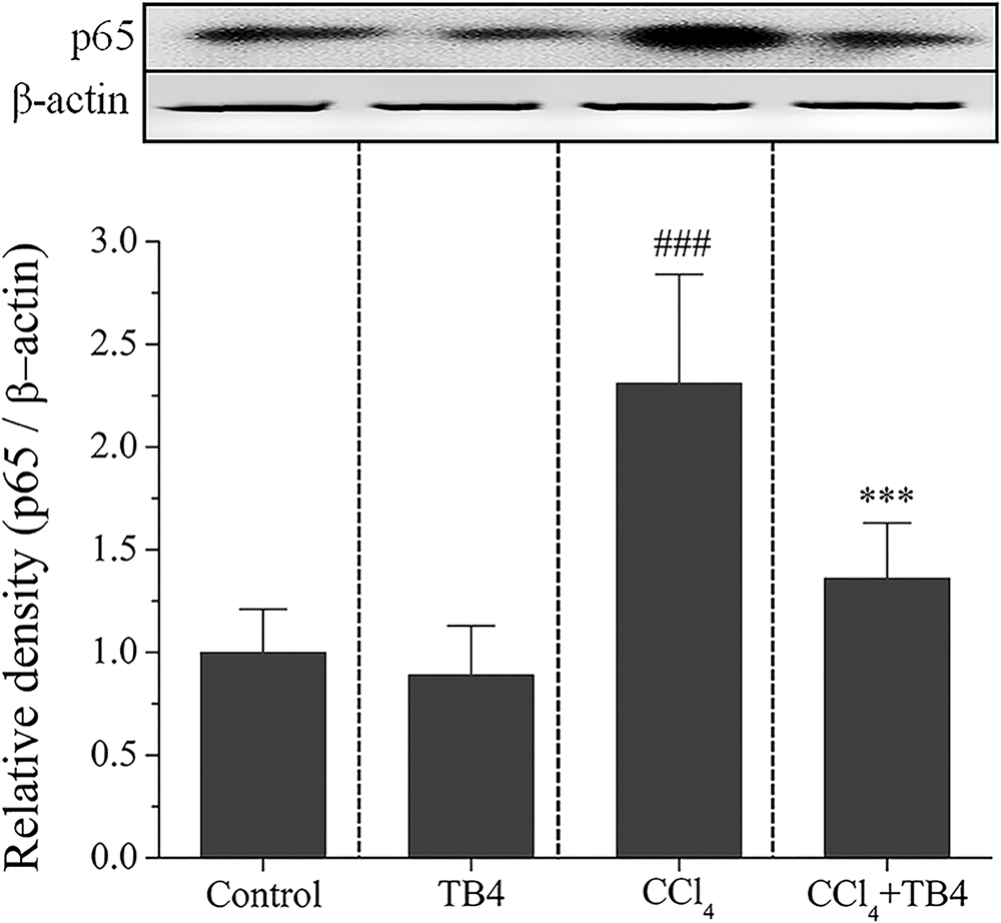
Western blot analysis of nuclear factor-κB (NF-κB) p65 protein in response to CCl_4_ and thymosin β4 (TB4) treatments. Six mice liver samples per group were analyzed by Western blot assays. Here showed the representative Western blot results and the semi-quantification results from Image J analysis. Data were expressed as mean ± standard error (n = 6). ^###^P < 0.01 vs. both control group and TB4 group; ***P < 0.05 vs. CCl_4_ group. β-actin was used as an internal control.

As shown in Fig. 5, CCl_4_ treatment significantly increased NF-κB p65 protein expression in the mouse livers (P < 0.01). However, the increase of p65 protein expression in the liver tissues was markedly suppressed by TB4 administration in CCl_4_-intoxicated mice (P < 0.05).

### Liver fibrosis

#### TB4 reduced CCl_4_-induced hepatotoxicity

**Figure 6:**
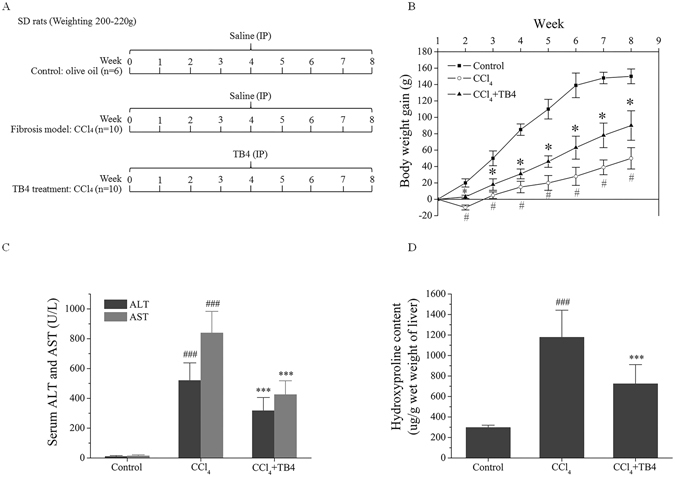
Experimental design and biochemical analysis. (**A**) Experimental design of the schedule for CCl_4_ and thymosin β4 (TB4). (**B**) Effect of TB4 on rat body weights; the body weight gains in CCl_4_-treated rats were significantly lower than those in control and CCl_4_ plus TB4 treated rats. ^#^P < 0.05 vs. control group; *P < 0.05 vs. CCl_4_ group. (**C**) Effect of TB4 on serum alanine aminotransferase (ALT) and aspartate aminotransferase (AST) activities in rats. ^###^P < 0.05 vs. control group; ***P < 0.05 vs. CCl_4_ group. (**D**) Effects of TB4 on hydroxyproline contents in rat livers. n = 6, control group; n = 10, CCl_4_ group; n = 10, CCl_4_ + TB4 group. ^###^P < 0.05 vs. control group; ***P < 0.05 vs. CCl_4_ group.

The SD rats were divided into three groups: control group, fibrosis model group, TB4 treatment group. Rats received eight-weeks of treatments as demonstrated in Fig. 6A. The body weights of all the rats were measured weekly and prior to scarification. As indicated in Fig. 6B, rats from the control group presented normal body weight gains from 220 g to 370 g over eight weeks. Whereas, the body weights of rats from fibrosis model group were significantly lower than those in control group. However, TB4 administration significantly prevented CCl_4_-induced body weight loss when comparing control group with fibrosis model group (Fig. 6B). In addition, CCl_4_-induced remarkable increases in serum ALT and AST activities were also significantly reduced by TB4 (Fig. 6C).

#### TB4 attenuated CCl_4_-induced hepatic fibrosis

**Figure 7:**
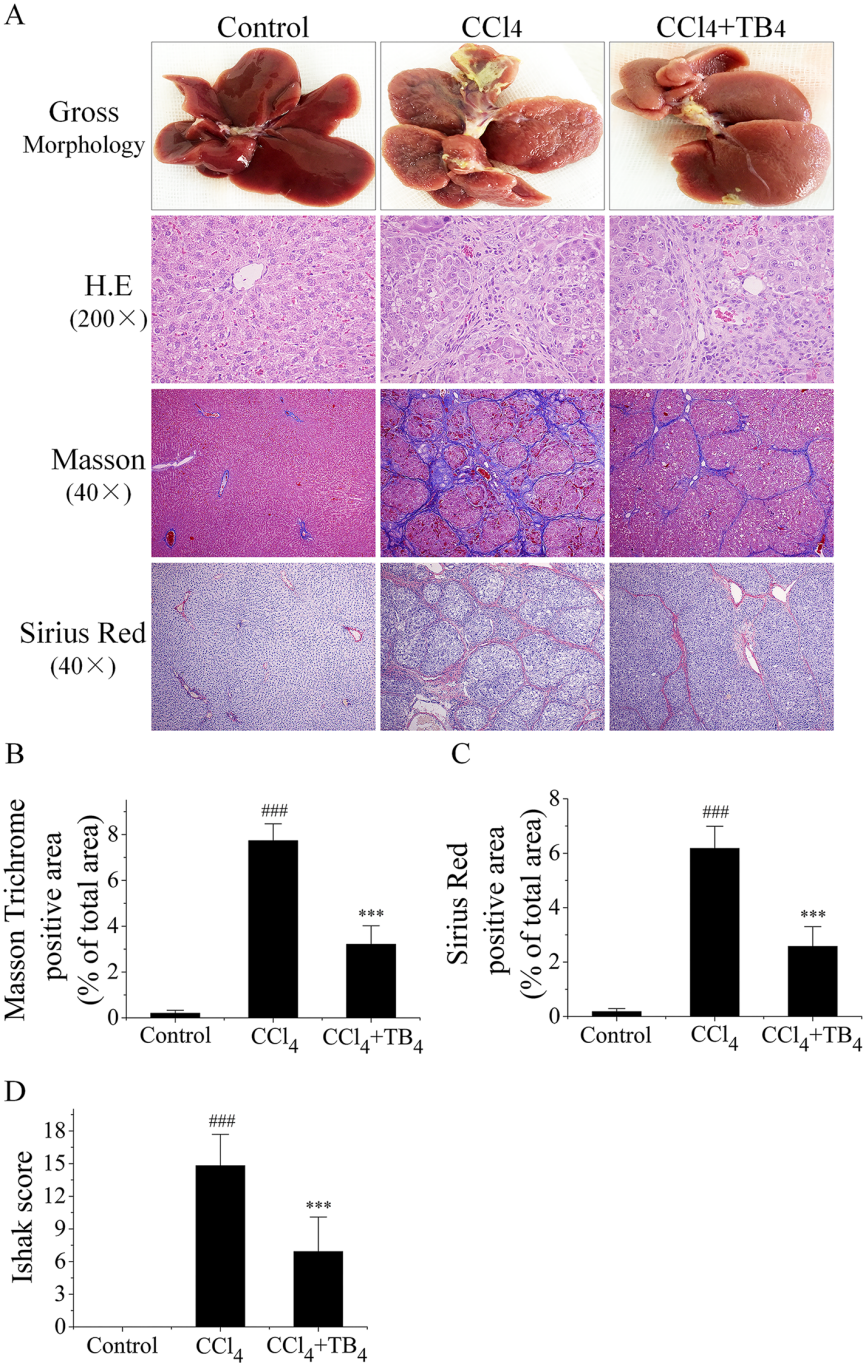
Effects of TB4 on liver fibrosis in rats. (**A**) Representative results of macroscopic appearance and liver histology staining of hematoxylin-eosin (H.E), Masson and Sirius Red were shown. Original magnification was indicated on the figure. (**B**) Semi-quantitative analysis of Masson trichrome staining results. (**C**) Semi-quantitative analysis of Sirius Red staining results. (**D**) Semi-quantitative analysis of liver fibrosis and inflammation using Ishak scoring system. n = 6, control group; n = 10, CCl_4_ group; n = 10, CCl_4_ + TB4 group. Two liver sections were analyzed for each animals. ^###^P < 0.05 vs. control group; ***P < 0.05 vs. CCl_4_ group.

Collagen deposition, one hepatic fibrosis marker, was determined and represented by hepatic hydroxyproline content. As shown in Fig. 6D, hydroxyproline contents in the liver tissues were significantly increased in CCl_4_-treated rats from fibrosis model group as compared with those from control group. However, TB4 treatment markedly decreased the hydroxyproline contents in CCl_4_-treated rats. Checks of macroscopic appearances of the livers showed that in comparison to normal livers in control group with a regular and smooth surface, the livers in CCl_4_-treated fibrosis model group were puffy, stiff, and acquired an irregular and granular surface. However, treatment with TB4 remarkably promoted the recovery of CCl_4_-damaged liver structure as shown in Fig. 7A.

H.E staining results showed that livers from the control group exhibited a normal lobular architectures, whereas livers from fibrosis model group exhibited damaged lobular architectures, severe vacuolar degeneration of hepatocytes, large fibrous septa, pseudo-lobule formations and inflammatory cells infiltration which were dramatically ameliorated by TB4 treatment (Fig. 7A). These results were further confirmed by Masson and Sirius Red stainings (Fig. 7A,B and C). Masson and Sirius Red stainings demonstrated that liver tissues from control group showed few collagen deposition, whereas those from CCl_4_-treated fibrosis model group presented dense fibrous septa and increased deposition of collagen fibers. Semi-quantitative analysis of the liver injury, fibrosis and inflammation using Ishak scoring system also showed that TB4 treatment significantly reduced CCl_4_-induced liver inflammation, injury and fibrosis (Fig. 7D). All these results provided evidences supporting the protective activities of TB4 against CCl_4_-induced hepatic fibrosis.

#### TB4 inhibited CCl_4_-induced oxidative stress and inflammation in the livers

**Figure 8:**
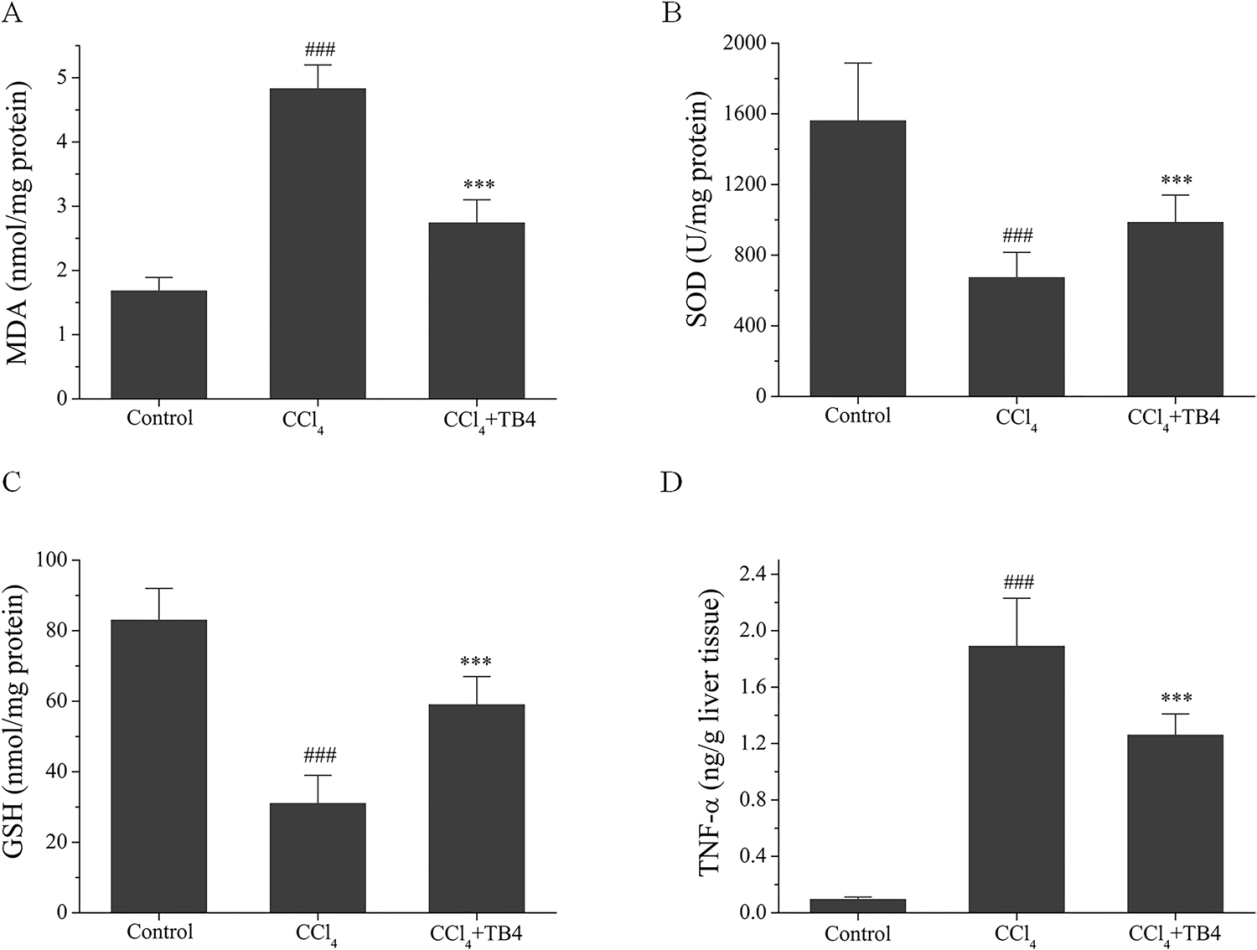
Effect of exogenous thymosin β4 (TB4) on oxidative stress parameters (**A**–**C**) and inflammatory cytokine TNF-α (**D**) in the livers of CCl_4_-treated Rats. n = 6, control group; n = 10, CCl_4_ group; n = 10, CCl_4_ + TB4 group. ^###^P < 0.05 vs. control group; ***P < 0.05 vs. CCl_4_ group.

**Figure 9:**
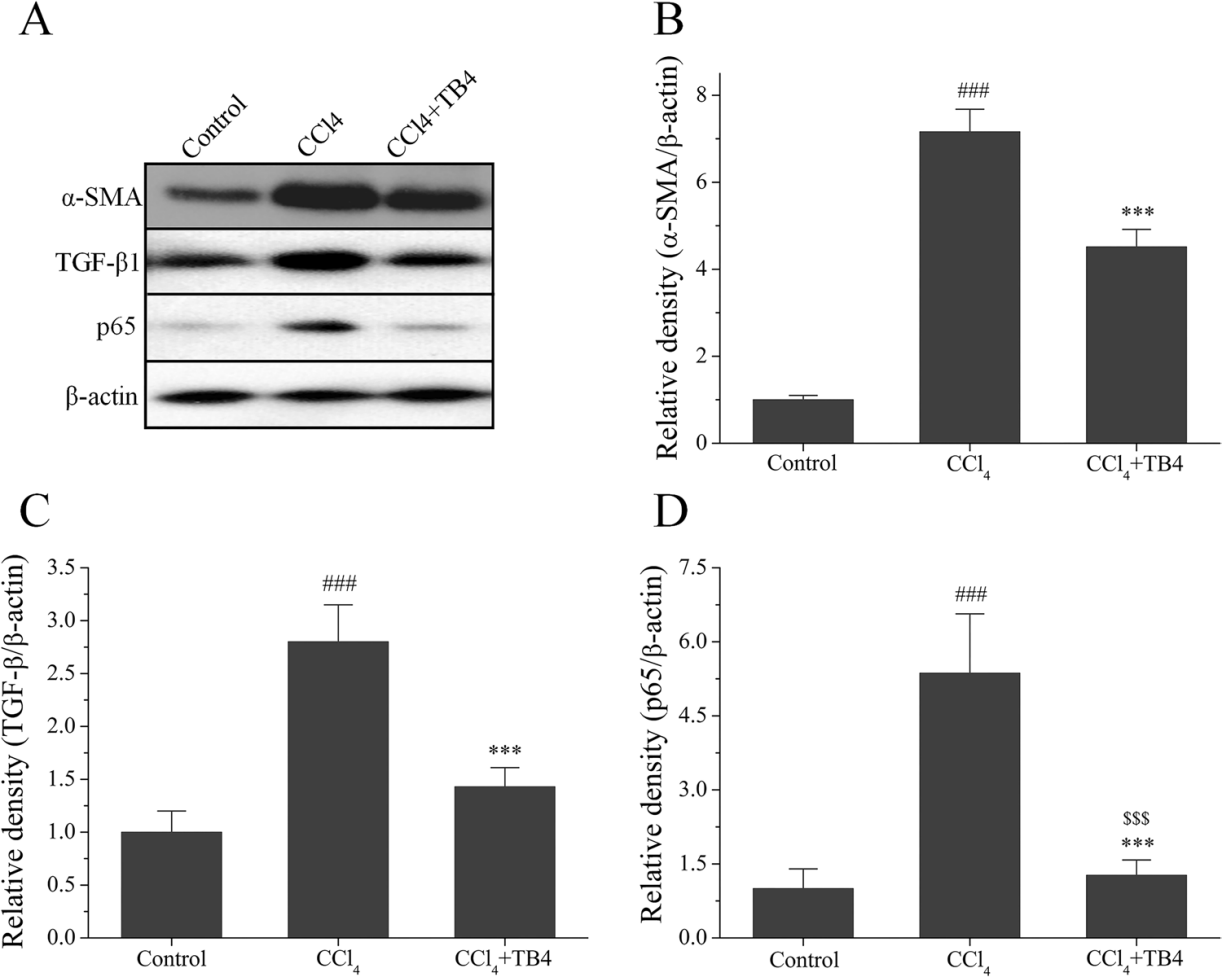
Western blot analysis of α-SMA, TGF-β1 and p65 in rat livers. Representative Western blot results (**A**) and semi-quantifications of the Western blot bands by Image J software were shown (**B**–**D**). Data were expressed as mean ± standard error of 6 rats per group. ^###^P < 0.01 vs. control group; ***P < 0.05 vs. CCl_4_ group; ^$$$^P > 0.05 vs. control group. β-actin was used as an internal control. (Some of the gels/blots in this figure were cropped from original full-length films which were illustrated in the supplemental information file).

As shown in Fig. 8A, MDA, a marker of lipid peroxidation, was remarkably increased in the livers from fibrosis model group as compared with control group (P < 0.01). On the contrary, the antioxidants SOD activities and GSH levels were markedly decreased in the livers from fibrosis model group in comparison to control group (Fig. 8B and C) (P < 0.01 for both). However, all these changes induced by CCl_4_ were significantly suppressed by TB4 treatment (P < 0.05). Pro-inflammatory cytokine, TNF-α, was up-regulated by CCl_4_ in the liver tissues as compared with control group (P < 0.01), which was greatly reversed by TB4 (Fig. 8D, P < 0.05). Furthermore, Western blots showed that expression of nuclear factor-κB p65 protein, a key player in inflammation, was remarkably increased in CCl_4_-treated fibrosis model group and was greatly reduced in TB4 treatment group (Fig. 9A and D).

#### Influence of TB4 on expressions of fibrosis markers of α-SMA and TGF-β1 after CCl_4_ administration

Expression of α-SMA and TGF-β1 in the liver tissues were determined by Western blots (Fig. 9). As indicated in Fig. 9, both α-SMA and TGF-β1 expressions were significantly up-regulated by CCl_4_ treatment in fibrosis model group compared with control group, and were significantly reduced by TB4 in TB4 treatment group in comparison to fibrosis model group.

## Discussion

Recent studies have report that TB4 is associated with fibrosis in several organs^28–31^, and more eyeballs of researchers are attracted by the role of TB4 in liver fibrosis^32^. Several studies illustrate that hepatocytes and hepatic stellate cells (HSCs) in the livers all express TB4 endogenously^33, 34^. The endogenous TB4 expression in HSCs is up-regulated in carbon tetrachloride (CCl_4_)-induced liver fibrosis^34^. In bile duct ligation (BDL)-induced liver fibrosis model endogenous TB4 expression is down-regulated in the fibrotic liver tissues^35^. In cultured human HSCs exogenous TB4 treatment inactivates HSCs through down-regulating PDGF-beta receptor expression and inhibiting PDGF-dependent phosphorylation and binding of AKT to actin^23, 36^. In LX2 (HSCs cell line) cell exogenous TB4 treatment inhibits its proliferation^35^. All these findings lead to a speculation that TB4 might be involved in the process of liver fibrogenesis^32^ and might be a promising target to treat liver fibrosis as exogenous TB4 has been proved to exhibit anti-fibrotic activities in kidney^28^ and lung^29–31^. However, so far, most of the key studies about TB4 in liver fibrosis are *in vitro* investigations^23, 34–36^ using primary cultures or cell lines of HSCs, a vital player in liver fibrogenesis^37^. Even so, conflicting evidences still exist at present. For example, Chen *et al*. reported that depletion of endogenous TB4 by siRNA activated HSCs *in vitro* ^35^; while Jung *et al*. proved that down-regulation of endogenous TB4 by siRNA inactivated HSCs *in vitro* ^34^. Moreover, even in some *in vivo* experiments conflicting results are also got. Results from Chen and his colleagues suggested that endogenous TB4 expression levels decreased during liver fibrogenesis^35^; however, reports from Jung *et al*. indicated that endogenous TB4 expression levels in livers increased during liver fibrogenesis^34^. So, at present, findings from those previous studies still can not clearly answer the question that whether exogenous TB4 exhibits inhibitory effects on liver fibrosis *in vivo*. In order to answer this question further *in vivo* experiments should be performed. In present study effects of exogenous TB4 were investigated in CCl_4_-induced acute mouse liver injury model and rat liver fibrosis model. Results showed that exogenous TB4 treatment markedly attenuated CCl_4_-induced acute mouse liver injury and chronic rat liver fibrosis. The mechanisms involved antioxidant and anti-inflammatory effects of TB4.

CCl_4_, a potent hepatotoxic agent, has been widely used to establish animal model to study liver injury which was characterized by typical centrilobular necrosis and was similar to the hepatotoxicity in human^38, 39^. The present study showed that in the acute mouse liver injury model a single intraperitoneal injection of CCl_4_ remarkably increased serum ALT and AST activities, which suggested the presence of acute hepatotoxicity since under normal condition AST and ALT only existed in both cytoplasm and mitochondria of hepatocytes. Histopathological examinations also reflected the severity of acute liver injury induced by CCl_4_. However, all these changes were significantly attenuated by TB4, which agreed with the previous conclusions reported by other researchers^27^. Immunohistochemistry results in current study demonstrated that CCl_4_ treatment increased the expressions of α-SMA, a marker of activated HSCs, and pro-fibrotic cytokine TGF-β both of which were reduced by TB4 treatment. Besides, the current study also showed that TB4 inhibited CCl_4_-induced oxidative stress and inflammation in acute mouse liver injury model, which was identified by TB4-induced suppression on CCl_4_-induced increases in MDA and nitro-tyrosine levels, decreases in SOD activities and GSH levels, up-regulations of TNF-α, IL-1β and nuclear factor-κB p65.

Although TB4 is investigated in previous animal studies of acute liver injury, so far it has not been studied in animal models of liver fibrosis. The current study aimed to explore the action of exogenous TB4 in CCl_4_-induced rat liver fibrosis. CCl_4_-induced rat liver fibrosis resembled human liver fibrosis as regards to the pathological processes and characteristics such as fiber formation, inflammation, regeneration and spontaneous recovery from fibrosis after removal of the toxic factor^40^. In current study rats were repeatedly exposed to CCl_4_ injection twice per week for eight weeks to induce liver fibrosis which were identified by increases in hepatic hydroxyproline contents and serum ALT and AST activities. Macroscopic appearance examinations showed that CCl_4_ injection rendered irregular and granular surface in rat livers. H.E staining results showed that extensive fibrotic lesions were present in rat liver tissues after 8-weeks of CCl_4_ treatment. Masson and Sirius Red staining results demonstrated that CCl_4_ injection caused pseudo-lobule formations, dense fibrous septa and increased collagen deposition in rat liver tissues. However, all these changes induced by chronic exposure to CCl_4_ were remarkably attenuated by TB4 treatment.

Fibrosis is the main pathophysiological consequences of chronic liver injuries which is caused by many factors including virus, autoimmune diseases, drug/toxin, alcohol and nonalcoholic fatty liver diseases^41^. Hepatic stellate cells (HSCs) activation plays a central role in the process of liver fibrogenesis^42^. During the liver injury trans-differentiation occurs in quiescent HSCs and makes quiescent HSCs activated. After activation HSCs acquire myofibroblast-like phenotypes with long processes and lose cytoplasmic lipid droplets. Furthermore, activated HSCs are pro-fibrogenic and promote fibrous extracellular matrix (ECM) proteins productions and deposition leading to liver fibrosis^43^. Previous studies have indicated that TGF-β played an important role in liver fibrosis by inducing myofibroblast-like cells formation. Through binding the transmembrane receptor TGF-β activates Smad signaling pathway and up-regulates expressions of ECM proteins^44^. As aforementioned above, single CCl_4_ injection increased the expressions of pro-fibrotic cytokine TGF-β1 and α-SMA, a fibrosis marker mainly expressed in activated HSCs. However, immunohistochemistry assays showed that TB4 treatment significantly suppressed the increases in TGF-β1 and α-SMA induced by single CCl_4_ injection. Similar to those results from acute liver injury study, Western blots results from chronic CCl_4_ exposure-induced rat liver fibrosis study showed that repeated CCl_4_ injection-induced up-regulations of TGF-β1 and α-SMA were also reduced by exogenous TB4 administration.

Oxidative stress mediated by free radicals derived from CCl_4_ is one of the main factors leading to hepatic damages. It causes cell membrane damage and consequent leakage of hepatotoxic marker enzymes^39^. Cytochrome P450 enzyme is involved in the process of CCl_4_-induced liver damages^45^. In hepatocytes cytochrome P450 catabolizes CCl_4_ to produce highly reactive trichloromethyl radical (**·**CCl3) and peroxyl radical (OOCCl3) which subsequently lead to cellular damages by initiating lipid peroxidation and covalently binding to macromolecules^45^. In present study oxidative stress was monitored by detecting oxidative stress parameters including MDA, SOD, GSH and protein tyrosine nitration. MDA is a product of lipid peroxidation and is used as a marker for lipid peroxidation, a key feature of CCl_4_-induced liver injury^46, 47^. SOD is an antioxidant enzyme that scavenged the superoxide anions^48^. GSH is the most important reducing substance in the body and collaborated with GSH-dependent enzymes to eliminate reactive intermediaries by reacting with hydroperoxides. It acts as free radical scavenger and plays an important role in maintaining protein sulfhydryl groups^49^. Previous study showed that GSH was depleted in hepatotoxicity^50^. Protein tyrosine nitration is considered as another marker of oxidative stress^51^. In CCl_4_-treated animals protein tyrosine nitration is increased in the liver tissues^27^. The current study showed that in both CCl_4_-induced acute liver injury model and fibrosis model MDA levels were markedly increased, and GSH levels and SOD activities were significantly decreased. Tyrosine nitration was increased in acute liver injury and was not detected in liver fibrosis at present. Administration of TB4 to CCl_4_-intoxicated animals significantly decreased MDA and nitro-tyrosine levels, but increased GSH levels and SOD activities in the liver tissues. These results indicate that TB4 harbors activities against oxidative stress induced by CCl_4_.

Besides oxidative stress, inflammation is also another important factor propagating CCl_4_-induced hepatotoxicity^52^. Numerous studies report that oxidative stress induced by CCl_4_ activate Kupffer cells which produce pro-inflammatory cytokines including TNF-α and IL-1^53–56^. TNF-α is considered as the main endogenous deleterious player in experimental liver injury model for its direct cytotoxicity and capacity to initiate inflammation cascades^57, 58^. IL-1 is another important inflammatory mediator and takes part in the progression from liver injury to fibrosis^59^. Blocking IL-1 through IL-1 receptor antagonist protects mice from CCl_4_-induced liver damage^60^. Many previous studies reported that pro-inflammatory cytokines production were regulated by nuclear factor-κB (NF-κB) signaling pathway^61, 62^. What’s more, previous study also demonstrated that in CCl-_4_ induced liver injury model inflammatory cytokines production were strongly correlated with the activity of NF-κB signaling pathway^63^. In our present study results indicated that CCl_4_ insults up-regulated the expressions of TNF-α, IL-1β and NF-κB p65 protein in the liver tissues. However, exogenous TB4 administration significantly suppressed the increases induced by CCl_4_. All these findings suggest that TB4 might exert hepatoprotection against CCl_4_ insults through inhibiting inflammation.

Taken together, findings in current study suggest that TB4 might prevent CCl_4_-induced acute liver injury and subsequent fibrosis through alleviating oxidative stress and inflammation. However, protective effects of TB4 on organ injury and fibrosis have been investigated by many other previous studies^28, 30, 31, 64–67^. Conte E. *et al*. reported that TB4 treatment attenuated bleomycin-induced lung injury and early fibrosis in mice^29, 30, 67^. TB4 also provides protection against renal injury and promotes renal repair during fibrosis^28, 64–66^. Reyes-Gordillo K. *et al*. reported that TB4 inhibited CCl_4_-induced acute liver injury in rats^27^, and our present results confirmed their findings. Previous researches and our present study all showed that inhibition of oxidative stress and inflammation were involved in the hepatoprotective activity of TB4^65, 67^. Nonetheless, more works should still be done to further elucidate the molecular mechanisms underlying TB4 actions. For example, since ac-SDKP, a degradation product of TB4, is anti-fibrotic in many organs including liver and kidney^68–72^, whether it is through ac-SDKP up-regulation that TB4 exerts anti-fibrotic activity in the liver? As TB4 could also modulate matrix metalloproteinases (MMPs) expressions in other organs or tissues^73, 74^ and MMPs play an important role in liver fibrosis^75^, then how does TB4 regulate MMPs expressions or activities in the liver tissues, and what are the significances of its regulation on MMPs in protection against liver injury and fibrosis? Clear answers for these questions warrant further investigations.

## Materials and Methods

### Ethics statement

This study was approved by the Institutional Ethics Committee for Animal Care and Use at Tianjin Medical University (Ethic No. TMUaMEC2015003). All animals received human care according to the Guide for the Care and Use of Laboratory Animals (Institute of Laboratory Animal Resource, 1996, Nat. Acad. Press).

### Chemicals and reagents

Thymosin β4 (HPLC > 98%) was purchased from GL Biochem. (Shanghai) Ltd and dissolved in 0.9% saline. α-SMA (Cat: CBL171) and β-actin (Cat: MAB1501), nitro-tyrosine (N-Tyr) (Cat: 05–233) antibodies were bought from Millipore (Darmstadt, Germany). P65 (Cat: 8242) antibody was purchased from Cell Signaling Technology (Denver, Colorado, USA). TGF-β1 (Cat: 18978-1-AP) antibody was bought from Proteintech (Wuhan, China). TNF-α (Cat: ab6671) and IL-1β (Cat: ab2105) antibodies were purchased from Abcam (Shanghai, China). Secondary antibodies against rabbit (Cat: 111-035-003) and mouse (Cat: 115-035-003) were obtained from Jackson ImmunoResearch (Baltimore, Maryland, USA). Immobilon enhanced chemiluminescence (ECL) detection reagent (Cat: WBKLS0500) was purchased from Millipore (Darmstadt, Germany). All other reagents were of analytic grade.

### Animals, treatments and groupings

Male Balb/c mice (6 weeks old, weighing ~20 g) and Sprague-Dawley (SD) rats (6–8 weeks old, weighing ~220 g) were bought from the Branch of National Breeder Center of Rodents (Beijing). All animals were maintained in specific-pathogen-free (SPF) environment (Experimental Animal Center at Tianjin Medical University) with a 12-h light/dark cycle and offered *ad libitum* access to food and water. Before experiments animals were acclimatized for a week under the SPF environment. At the end of the experiments animals were euthanized under pentobarbital anesthesia.

Acute liver injury was induced by a single intraperitoneal injection of 0.5 ml/kg.bw (bw, body weight) CCl_4_ in olive oil in Balb/c mice. Twenty-four hours later mice were sacrificed to collect blood and liver tissues for further analysis.

Rat liver fibrosis model was established through CCl_4_-indueced persistent chronic liver injury for eight weeks. CCl_4_ in olive oil was intraperitoneally administrated twice a week for 8 weeks. The dosage of CCl_4_ was 0.5 ml/kg.bw (bw, body weight).

Mice for acute liver injury induction were divided into four groups (n = 10/group) as followings: (1) Control group, mice in this group received nothing but equal volume of olive oil intraperitoneally; (2) TB4 group, mice in this group received intraperitoneal administration of both olive oil and 100 μg TB4/mouse/time; TB4 was administrated at 0 hour, 2 hours, 4 hours, 6 hours after olive oil injection; (3) CCl_4_ treatment group, mice in this group received only CCl_4_ in olive oil intraperitoneally to induce acute liver injury; (4) CCl_4_ + TB4 treatment group, mice in this group were treated with CCl_4_ and TB4 intraperitoneally; TB4 were administrated at 0 hour, 2 hours, 4 hours, 6 hours after CCl_4_ injection. The dose and time of TB4 were determined according to previous report^76^.

Rats for liver fibrosis induction were divided into three groups as followings: (1) Control group (n = 6), rats in this group received intraperitoneal injection of olive oil and saline; (2) Fibrosis model group (n = 10), rats in this group received intraperitoneal injection of CCl_4_ in olive oil and saline; (3) TB4 treatment group (n = 10), rats in this group were treated with CCl_4_ plus TB4; CCl_4_ was administrated as aforementioned; TB4 were intraperitoneally administrated once every three days at 1 mg/kg body weight. The dose and time of TB4 were determined according to previous study^27^.

### Biochemical assays

Serum ALT and AST activities, SOD activities and MDA, GSH levels in the liver tissues, were determined by commercially available detection kits (Nanjing Jiancheng Institute of Biotechnology, Nanjing, China) according to the manufacturer’s instructions. Hepatic hydroxyproline content was also measured using a detection kit from Nanjing Jiancheng Institute of Biotechnology (Nanjing, China) according to the manufacturer’s manual. The results were reported as microgram of hydroxyproline per gram of wet liver tissue. TNF-α was measured using a commercial ELISA kit (Cat: ab46070 and ab100747, Abcam, Shanghai, China). Mouse IL-1β ELISA kit were purchase from ThermoFisher Scientific (Cat: BMS6002, Shanghai, China).

### Histopathology

Liver tissues were fixed in 10% neutral buffered formalin, processed routinely, embedded in paraffin and then were cut into 4-μm thick sections. Liver sections were stained with hematoxylin-eosin (H.E) according to standard procedure for routine histological examination. Liver sections were stained with Masson’s trichrome and Sirius Red stains to estimate liver fibrosis. Liver fibrosis was semi-quantitatively analyzed using Image J free software (http://rsb.info.nih.gov/ij/).Stained liver slices were examined under a Nikon light microscope by an experienced pathologist. Liver injury and fibrosis were evaluated and scored through Ishak scoring system^77^.

### Immunohistochemistry

For immunohistochemistry, liver sections were deparaffinized and rehydrated. Endogenous peroxidase activities were blocked in 3% H_2_O_2_ for 10 min. Antigen was retrieved in citrate buffer (pH = 6.0) in a microwave oven for 15 min. Non-specific protein binding was blocked by BSA (5%). Then the sections were probed with specific primary antibodies against α-SMA (1:200), TGF-β1 (1:200), nitro-tyrosine (1:200), TNF-α (1:200) and IL-1β (1:200) overnight in a humidified chamber at 4 °C. After washing with PBS twice, the liver sections were incubated with a biotinylated secondary antibody. Then the immunoreaction was amplified with streptavidin–avidin–peroxidase complex. Liver sections were stained with diaminobenzidine (DAB) Chromogen for color development. At last, sections were lightly counter-stained with hematoxylin, mounted with mounting medium and examined under a light microscope by an experienced pathologist. Positive antigen stained brown against a blue hematoxylin background.

### Western blot analysis

Total protein samples were extracted from snap-frozen liver tissues (80–100 mg) of different groups using 1 ml RIPA lysis buffer (Pierce, Rockford, Illinois, USA) supplemented with 1 mM phenylmethylsulfonyl fluoride (PMSF) (Sigma-Aldrich, St. Louis, Missouri, USA), a protease inhibitor cocktail (Amresco, Solon, Ohio, USA) and phosphostop (Roche Diagnostics, Indianapolis, Indiana, USA). Total extracts were collected by centrifugation at 14000× g for 10 min at 4 °C. The concentration was determined using a BCA protein assay kit (Pierce, Rockford, Illinois, USA). Then protein samples were subjected to SDS-PAGE (15%) and transferred onto a 0.2-μm PVDF membrane (Millipore, Darmstadt, Germany). Membranes were blocked with 5% skimmed milk and incubated with specific primary antibodies against p65 (1:1000), α-SMA (1:1000), TGF-β1 (1:1000) and β-actin (1:10000) overnight at 4 °C. Membranes were incubated with horseradish peroxidase (HRP)-conjugated goat anti-rabbit or goat anti-mouse secondary antibody (1:20000) at room temperature for 1 hour. At last, protein bands were visualized using Immobilon enhanced chemiluminescence (ECL) reagents and imaged using a GelDoc XR System (Bio-Rad, Shanghai, China). Bands densities were analyzed using Image J free software (http://rsb.info.nih.gov/ij/).

### Statistical analysis

Statistical analyses were performed using SPSS software for Windows, version 21.0 (SPSS Inc., Chicago, Illinois, USA). Student *t*-test was employed to compare the differences between groups. P < 0.05 was considered as statistically significant.

## Electronic supplementary material

Suppelmentary Information
