# Supplementary material for: Effects of exogenous thymosin β4 on carbon tetrachloride-induced liver injury and fibrosis
Source: Sci Rep. 2017 Jul 19;7:5872. doi: 10.1038/s41598-017-06318-5 (PMC5517632; doi:10.1038/s41598-017-06318-5)
Supplement: Supplementary file 1 — Suppelmentary Information [file 41598_2017_6318_MOESM1_ESM.pdf]

# Effects of exogenous thymosin β4 on carbon tetrachloride-induced liver injury and fibrosis

Xiankui Li, Lei Wang, and Cai Chen

Supplemental Information

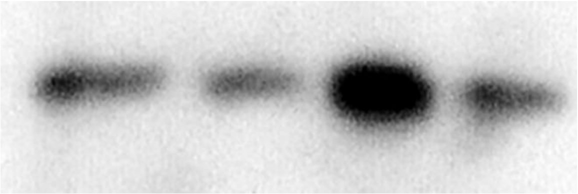

Blot results of p65 protein in Fig 5. We are so sorry that the original film can't be found. If it's necessary, we are willing to do this blot again to accommodate the requests of Scientific Reports.

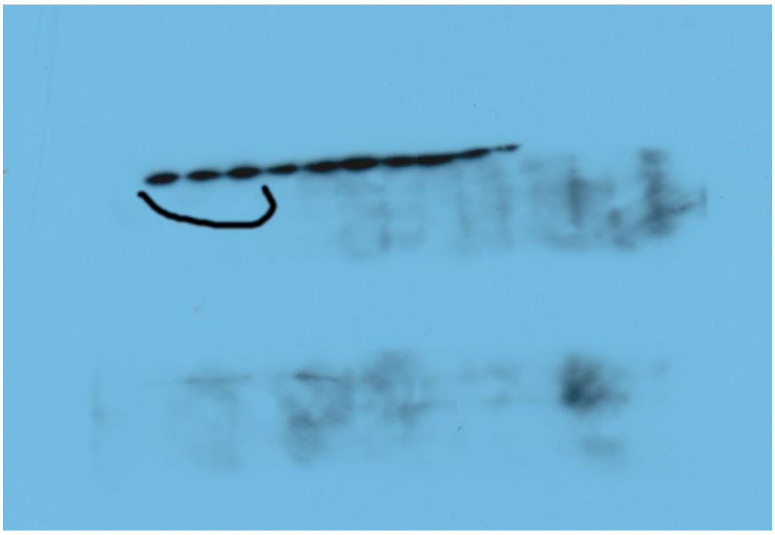

β-actin blots/gels in Fig. 9 were cropped from this original film. The left three bands were used in Fig. 9 and marked with a line in the film.

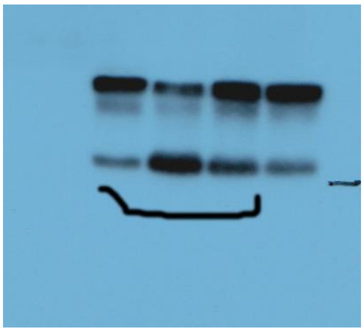

α-SMA blots/gels in Fig. 9 were cropped from this original film. The lower left three bands were used in Fig. 9 and marked with a line in the film.

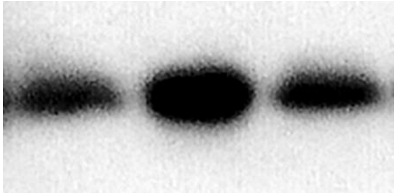

Blot results of TGF-β1 protein in Fig. 9. We are so sorry that we didn't find the original film. If it's necessary, we are willing to do this blot to accommodate the requests of Scientific Reports.

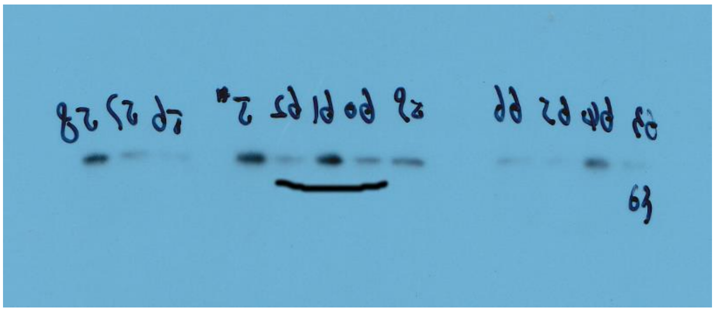

p65 blots/gels in Fig. 9 were cropped from this original film. The middle three bands were used in Fig. 9 and marked with a line in this film.
